# Technical Challenges of Utilizing Ground Tire Rubber in Asphalt Pavements in the United States

**DOI:** 10.3390/ma14164482

**Published:** 2021-08-10

**Authors:** Rouzbeh Ghabchi, Amir Arshadi, Musharraf Zaman, Ferrella March

**Affiliations:** 1 Department of Civil and Environmental Engineering, South Dakota State University, Brookings, SD 57007, USA; 2 Project Manager, AECOM, Chelmsford, MA 01824, USA; Amir.Arshadi@aecom.com; 3 School of Civil Engineering and Environmental Science, University of Oklahoma, Norman, OK 73019, USA; Zaman@ou.edu; 4 Used Tire Recycling Program, Oklahoma Department of Environmental Quality, Oklahoma City, OK 73102, USA; Ferrella.March@deq.ok.gov

**Keywords:** hot mix asphalt (HMA), ground tire rubber (GTR), wet process, dry process, rubberized asphalt, asphalt mix performance

## Abstract

At least 275 million scrap tires exist in stockpiles in the U.S. The practice of dumping scrap tires in landfills has been an environmental concern. To address this concern, many industries—and regional and national environmental protection agencies—have taken major initiatives to recycle scrap tires. One of the major uses of recycled scrap tires is in crumb rubber products, including rubberized asphalt. Rubberized asphalt is produced by blending ground tire rubber with asphalt to beneficially modify its properties for highway construction. The ground tire rubber (GTR) can be used either as part of the asphalt rubber binder (also known as asphalt rubber), seal coat, cap seal spray, joint and crack sealant or as substitute aggregate (rubber-modified asphalt concrete). Therefore, the largest single market for GTR is asphalt rubber, which consumes approximately 12 million tires, annually. Currently, several Departments of Transportation (DOTs) in the U.S. do not allow use of GTR in asphalt mixes. This is partly due to lack of information, laboratory test data and specifications or special provisions on the use of GTR in asphalt pavements. The current study was undertaken to summarize the available wealth of knowledge, identify research needs, and document the major findings of previous pertinent studies focused on GTR use in asphalt. Significant study findings—consisting of laboratory test results, field observations, and common practices—were documented, including: the use of GTR in asphalt mixes, wet and dry processes, characterization of hot mix asphalt (HMA) containing GTR and GTR performance when combined with virgin materials. In order to promote successful use of GTR, it is imperative to help DOTs develop specifications/special provisions for utilizing rubberized asphalt by collecting data, common practices and specifications utilized by other state DOTs. As a part of this effort, we conducted a survey of construction specifications used by different DOTs that currently allow the use of GTR in asphalt. Since some DOT practices are not readily available in the open literature, this survey proved to be an effective tool for gathering data on the current practices, methods and specifications associated with DOT use of GTR in asphalt pavement.

## 1. Introduction

Annual generation of scrap tires in the United States increased from 200 million in the 1980s to 300 million in the 2010s and has increased steadily each year since because of the projected growing number of vehicles [1,2,3]. Approximately 80% of all annually generated scrap tires in the United States are recycled or reused as furnace fuel, landfill cover, athletic field infill, mulch and in agricultural and structural engineering applications (Willis et al., 2012).

Over the past fifty years or so, when the idea of using scrap tire rubber in asphalt pavements arose, it seemed that the push to do so was primarily a means of getting rid of piles of scrap tires, as they were visually offensive and a health/fire hazard. In one of the earlier GTR paving efforts, recycled tire rubber was added in place of asphalt binder using a system called Plus Ride. Larger-sized recycled rubber (often finer than 0.6 mm) was added to hot mix asphalt (HMA) much like an aggregate, and the resulting mix was placed and compacted like a standard hot mix asphalt. This early rubberized asphalt paving effort had elevated levels of failure because of mix design problems (including insufficient binder, no production heating control, no dwell time control for produced mixes, compaction issues, material handling issues and nonrepresentative distribution of rubber in aggregate), but when finer crumb rubber was combined in asphalt binders for use in asphalt production, researchers found that rubber-modified asphalt pavements performed at least as well as standard HMA pavements [4]. Although the use of recycled rubber in pavements was desirable from an environmental sustainability standpoint, the cost of rubber modification was typically higher than standard modified asphalt pavements. Only with a well-established quality control system in place were rubber asphalts able to show a field performance better than or equal to that of conventional mixes.

Crumb rubber (CR), or ground tire rubber (GTR), is recycled tire rubber that has been ground into fine particles that can be used as an asphalt modifier. In the 1990s, a Federal Highway Administration (FHWA)/United States Department of Transportation (USDOT) national mandate for the use of rubber in asphalt focused on what has been called either the wet process or terminal blend asphalt. In those methods, finer mesh crumb rubber is introduced into liquid binder (often with other additives), followed by cooking and digestion of the rubber into the binder before its use in asphalt production. The rubber mandate was a part of the Intermodal Surface Transportation Efficiency Act (ISTEA) of 1991 [5]. As the mandate was rolled out, a lack of clear standards and deficient preparation for technology deployment (e.g., insufficient user training) created a number of problems in the field, ultimately resulting in the cancellation of the rubber use requirement by FHWA/USDOT. However, rubber-related enhancement of mix performance was observed by engineers and researchers [5,6,7,8,9,10,11,12,13]. It was shown that asphalt rubber in dense-graded mixes could reduce the asphalt layer thickness by 20–50% without affecting its performance [14]. In a research project conducted in Brazil, it was found that having 15% rubber in the HMA overlay binder reduced crack development by 5–6 times, compared to mixes using conventional asphalt binder [15].

Mashaan et al. [16] presented an overview of the use of rubber-modified binders in HMA. Their findings indicated that the use of crumb rubber modifier not only reduced pollution problems but also resulted in safer and smoother roads. They also found that crumb rubber modifier improved rutting resistance and produced pavements with superior durability. In a recent study conducted in India, it was shown that application of crumb rubber modifier in HMA improved the rutting resistance and durability of the pavement [17]. Crumb rubber modification improves the temperature-susceptibility of the asphalt binder and generally bumps up both high and low performance grade (PG) temperatures [18]. In terms of pavement performance, wet process crumb rubber-modified (CRM) mixes have the potential to resist crack propagation better than other polymer-modified and conventional mixes [18]. Fraser [19] showed that modification of asphalt binder with GTR increased the stiffness, maximum load capacity and strength of the resulting asphalt mix compared to regular HMA. In another recent study, it was found that asphalt rubber modification improved the binder performance in terms of rutting and fatigue resistance by increasing the complex shear modulus and storage modulus and decreasing the phase angle [20]. Air voids and voids in mineral aggregates are two key parameters that influence the rutting performance of wet process CRM mixes [21]. 

Laboratory and field studies on a gap-graded mix with 20% crumb rubber (by the weight of binder) indicated that use of high content of crumb rubber reduced the noise from tire–pavement interaction by 2.5 dB(a) for vehicles driven at 50 mph [22]. In another research study—conducted on a two-lane road located in Spain—it was concluded that a wet process CRM mix containing 20% rubber (by the weight of binder) was quieter than the reference mix by about 1 dB(a) at 32 mph [23]. Another study [24] reported that the technique used for grinding tires largely affected its surface area and the properties of asphalt binders containing it. Rubber Pavements Association (RPA) conducted a noise study and found that the use of tire rubber in open-graded mixes reduced tire noise by at least 50%.

Shirini et al. [25] investigated the effect of crumb rubber modification on porous asphalt performance. They indicated that, although crumb rubber modification enhanced the resilient modulus, skid resistance, resistance to moisture-induced damage, and rut resistance of the asphalt mix, it had a negative effect on mix permeability. It was concluded that crumb rubber content of 10% was the most effective dosage with respect to the overall performance of a CRM mix.

Charles H. McDonald from Arizona, known as the inventor of asphalt rubber, developed the McDonald process (also called the wet process) for production of asphalt rubber [26]. Arizona, California, and Texas are the leading states in asphalt rubber utilization. Together, these states recycled almost 36 million scrap tires in asphalt pavement applications from 1995 to 2001 [2].

The number of states where tire rubber is routinely used in asphalt pavement applications has been increasing steadily [27]. In a recent study, and as a part of FHWA-HIF-20-043 [28], it was reported that use of GTR in asphalt binder and mixes as an accepted practice in the asphalt pavement industry constitutes about 12% of the total GTR global market. It is estimated that about 17% of the produced scrap tires in the U.S. in 2017 were used in GTR-modified asphalt binders [28]. Furthermore, GTR was reported to be used in asphalt binders at minimum rates varying from 10% in California and Louisiana to 20% by weight of asphalt binder in Arizona [28].

The primary applications of crumb rubber-modified asphalt binder by the pavement industry include crack and joint sealants, binders for chip seals, interlayers, HMA and membranes. Chip seals can be placed on the pavement surface or as a stress-absorbing membrane interlayer (SAMI) placed between pavement layers. It was shown that the application of GTR-modified binders in interlayers and chip seals provided a longer service life in comparison with conventional asphalt binders without any GTR [29]. Gap-, open-, and dense-graded GTR-modified HMA mixes can also be successfully placed as surface course [2,30]. The vast majority of rubber use in California is in HMA, which includes full depth and overlays.

While the quality and performance of CRM mixes largely depends on the material type, recycling technologies, and processes applied in their production, a number of potential drawbacks associated with CRM have been reported in the literature. For example, there has been concern pertaining to storage stability of CRM asphalt binders [31]. Additionally, a lower stiffness in CRM mixes and an increase in affinity to moisture damage in wet-process CRM mixes compared to their polymer-modified asphalt (PMA) counterparts has been reported [32]. Furthermore, some studies suggested that performance of the CRM mixes after recycling could be negatively affected by changes in mix volumetrics and asphalt binder rheology [33]. It should be noted that each study had its own limitations and, depending on the materials and techniques used, their findings could differ completely from one to another. Therefore, those results may not be taken out of their contexts and generalized for all types of asphalt mixes containing GTR. Table 1 summarizes key findings of selected studies.

## 2. Study Objectives

This study was conducted to summarize the available wealth of knowledge, identify research needs, and document the major findings of previous pertinent studies. In addition to using the open literature as a resource, a survey of construction specifications used by different DOTs which allow the use of GTR in asphalt was conducted. Since DOT practices are generally not available in the open literature, this survey was found to be an effective way for gathering data on the current practices including the methods, special provisions, and specifications associated with the use of GTR in asphalt pavement.

## 3. GTR-Modified Binders and Mixes

Crumb Rubber Preparation: Crumb rubber additions to asphalt can vary based on rubber chemistry, preparation processes and the presence of belt residuum. Although the make-up of the tires varies depending on their type (truck tires have more natural rubber content than passenger car tires) and manufacturer, the basic components of different tires are very similar. The general experience with rubberized asphalt indicates that slight variations in the amount of natural and synthetic rubber do not cause differences in performance of GTR-modified binders [49]. This is because GTR does not melt or devulcanize in asphalt binders; thus, the chemistry variations of tires don’t impact pavement performance. The presence of fiber and metal debris from tire belts can negatively impact pavement performance. In order to use tires as a binder modifier or a mix additive, steel and fiber should be removed.

One of the factors that can affect the performance of GTR-modified binders is the method used for grinding the tire rubber [2]. Grinding methods include (but are not limited to) ambient grinding, cryogenic grinding, granulation and shredding [50]. It should be noted that the two primary grinding methods are ambient and cryogenic grinding [49].

Ambient grinding occurs at or above ordinary room temperature and involves grinding the scrap tire to provide irregularly-shaped particles with large surface areas. Higher surface areas promote faster and larger interactions between crumb rubber and binders. In the cryogenic process, liquid nitrogen is used to freeze the rubber and increase its brittleness and then a hammer mill is used to shatter the frozen rubber into smaller particles. This granulation process uses a revolving steel hammer to shred the scrap tire to cubical particles with low surface areas. The shredding process reduces the scrap tires to pieces smaller than 6 in^2^ prior to granulation of ambient grinding.

Binder and Mix Modification with Rubber: Two distinct approaches are used for incorporating the ground tire rubber into asphalt pavements: the (i) wet process and the (ii) dry process. The modified asphalt binder obtained from the wet process is termed asphalt rubber, and asphalt made using the dry process is called rubberized asphalt [51]. The term rubber asphalt is used when the rubber content by weight of the asphalt binder is 15% or higher. The term rubberized asphalt is used if the rubber content constitutes less than 15% of the asphalt binder’s weight. It should be noted that each of these processes produce GTR-modified asphalt mixes with different performance characteristics. In order to make the right choice on the type of process, understanding their differences is very important. In addition, testing and inspections are needed with each of these processes to ensure their success.

### 3.1. Wet Process

As noted above, the wet process for rubberized asphalt involves mixing of recycled tire crumb rubber with an asphalt binder in high temperature (176 to 226 °C), followed by a period of cooking and digestion (hours or days) and continued agitation in order to keep the crumb rubber suspended in the binder [14]. Unlike polymers, the recycled tire rubber does not become a near-integral part of the binder. The crumb rubber used in the wet process has a higher density than the binder, allowing the rubber and binder to separate if not maintained in a turbulent environment. During heating, the crumb rubber will soften and swell because of surface absorption of lighter binder components in the surface pores of the rubber [52,53,54]. The swelling process is caused by a selective removal of the asphalts’ lighter ends from the binder while adding swollen crumb rubber to the mix matrix. This increases the viscosity of the binder, stiffens the mix and increases resistance to permanent deformation (rutting). The presence of softened rubber grains in the mix also makes the asphalt more flexible, thus increasing resistance to various forms of cracking [55]. In addition, dissolving rubber in asphalt binder increases its viscosity, allowing higher binder content to be used in the mix. Theoretically, this leads to asphalt mixes with improved fatigue resistance and durability [56]. Extended heating time of rubber crumbs is expected to weaken the pore structure in rubber, resulting in the release of binder light ends and tire processing oils, thus reducing the dynamic viscosity of the binder.

Although some companies using the wet process have suggested that there might be some sort of material chemical exchange between the binder and the crumb rubber during heating and devulcanization, the assertion is controversial. The bulk of vulcanized tire rubber cannot melt at asphalt plant temperatures (149–205 °C), but will decompose/oxidize at a higher temperature (>316 °C) over time. Xiao et al. [57] suggested that there was a modest mass loss from crumb rubber over extended cooking periods in an asphalt binder, but Peralta et al. [55] suggested that mass loss was due to splitting of the crumb rubber into finer particles and contribution of processing oils from the tire rubber. It was also observed that the depolymerization potential of recycled crumb rubber is quite low, primarily because of the strength of bonding due to vulcanization [55].

An important question to be answered is: if the heated binder does not materially polymerize the recycled rubber, what is the binder modification mechanism for crumb rubber in asphalt? There is ample evidence of some binder light ends (Maltenes) uptake in the surface pores of rubber grains when heated [54,55]. Outcomes of a research study conducted at the National Center for Asphalt Technology (NCAT) clearly suggested that, for shorter cooking times (characteristic of dry process and Arizona wet process rubberized asphalt), the primary interaction between rubber and binder is mechanical in nature, not chemical. This uptake process causes two physical/mechanical changes in the asphalt binder and mix: (i) an increase in binder viscosity and (ii) a swelling of the crumb rubber. These changes influence the performance of the binder and any produced asphalt mix. The rubber additions and withdrawal of light ends increase the G*/sin δ of the binder, which is an indicator of rutting resistance. Therefore, rubberized pavements are more resistant to permanent deformation. The swelling of the crumb rubber also adds a larger flexible mass to the asphalt mix matrix, providing an increased degree of pavement flexibility (increasing the cracking resistance of the rubberized pavement). Mechanical changes aside, there appears to be little likelihood of any chemical exchanges between the rubber and the binder during blending, transporting and mixing for hot and warm mix production. Finally, there is a second change in the character and performance related to the physical presence of crumb rubber in the asphalt matrix: a typical 8 lb. crumb rubber dose rate in an asphalt mix adds roughly 40 million individual crumbs of rubber per ton of asphalt mix. In the event that any cracking might begin in the asphalt, each crumb of rubber can help to pin or deflect cracks, stopping or slowing crack propagation.

Also, it should be noted that the properties of asphalt rubber are influenced by base binder composition, blending time, temperature, amount/size of ground tire rubber and the grinding method used [50,51,58,59,60,61,62]. It has been suggested not to use polymer-modified binders for asphalt rubber as the polymer-rubber interaction affects the durability of asphalt mixes [63]. There are two methods for preparing wet process asphalt rubber: (i) terminal blending and (ii) on-site blending (Table 2).

#### 3.1.1. On-Site Blended Crumb Rubber-Modified Asphalt

On-site blending in wet process is entirely carried out in the asphalt plant, which is equipped with special equipment such as blending and storage tanks with heater systems. Wet process consists of blending the bulk GTR transported to the asphalt plant and then blending them with the asphalt binder [28]. Blending the asphalt binder with the GTR is usually conducted at temperatures ranging from 175 to 190 °C for 30 to 60 min to achieve swelling of GTR and proper physical reaction between binder and rubber phases. Right after this process, the GTR-modified binder is used in the asphalt plant, like regular asphalt binder, for production of asphalt mixes. According to Baumgardner et al. [28], in-place wet process normally allows 10–22% GTR (by asphalt binder weight) to be used. The maximum GTR size used in in-place wet process is 1.5 mm.

#### 3.1.2. Terminally-Blended Crumb Rubber-Modified Asphalt

Terminally-blended crumb rubber-modified asphalt is typically produced at an asphalt terminal. Crumb rubber is introduced into heated binders in a turbulent environment. The rubber is allowed to cook at high temperatures for hours or days under continuous agitation. Terminals routinely add chemicals to the rubberized binder in order to meet performance and quality specifications. The finished product is pumped into trucks or rail cars (with or without agitation capabilities on board). Like polymer-modified asphalt binders, the suspended rubber/asphalt solution is transported by truck or rail to the end user, where the modified binder is stored in a dedicated tank before use. Separation of rubber and binder during transportation in unagitated trucks is common and can lead to quality issues in the finished mix product. In addition, asphalt tankers do not always pump all of the settled rubber out of the tanker, and rubber clean-outs may be necessary before the tanker can be used again.

Xiao et al. [64] investigated the rheological properties of terminally-blended and laboratory-blended asphalt binders in terms of three aging states: unaged, short-term aged, and long-term aged. Their results indicated that the rheological properties of both binders were similar. A recent study on the long-term field performance and life cycle costs of pavement sections constructed with binder that was terminally-blended with GTR and/or poly styrene-butadiene-styrene (SBS) polymer modifier showed that both pavement sections had good performance after 10 years of service [65]. Laboratory studies on field cores extracted from asphalt pavement sections constructed using terminally-blended GTR and SBS polymer-modified binder exhibited similar fatigue resistance for both sections but GTR field cores showed slightly higher resistance to low temperature cracking and moisture-induced damage. Life cycle costs for the section constructed by using mixes containing GTR was slightly higher than the one constructed using polymer-modified mixes due to the higher initial cost of GTR mixes. Another life cycle cost analysis indicated that the widespread use of asphalt rubber has been cost-effective in Arizona and California [66].

Mix designs with rubber require differing virgin binder amounts than their polymer-modified counterparts. Since the rubber does not add binder equivalence, 5–10% additions of rubber to a mix design actually require the addition of virgin binder to compensate for the presence of rubber in the modified binder, as well as the limited amounts of binder absorption on the rubber surfaces.

Rubberized asphalt mixes can be significantly stickier than standard hot- and warm-mix asphalts depending on the amounts of rubber used. This can make the wet process crumb rubber-modified asphalt mixes more difficult to produce, transport and place/compact.

#### 3.1.3. Current Market for the Wet Process/Terminal Blend Rubber

Once the wet process crumb rubber-modified asphalt pavements were demonstrated to be an effective alternative to polymer modification, asphalt producers began to focus on the relative costs and benefits of rubber versus polymer modification. Users noted that higher levels of rubber addition required the use of special equipment and handling procedures because the rubber additions made the hot- and warm-mix asphalt stickier during material production, movement and placement/compaction. This required the development of special equipment and additives that would allow effective and efficient addition of rubber to asphalt. As wet process adoption began to expand, the technology was written into various state specifications (AZ, TX, CA). Most of the polymer modification of binders already occurred at various oil terminals, and when rubber was introduced, DOTs were effectively asking many terminals to introduce a rubberized asphalt product. With a pricing model that did not offer any performance or competitive advantage, the wet process or terminal blend rubber-modified asphalt has been somewhat successful where its use has been mandated or strongly encouraged by state DOTs.

The FHWA notes that some earlier cold climate failures have been tied to construction issues, but rubberized pavement designs should be effective in colder climates [49]. Wang et al. [67] reported that the resistance of rubberized asphalt to low-temperature cracking was affected by mixing temperature, mixing duration and rate. It was also found that, for an acceptable resistance to thermal cracking, production parameters should be selected carefully. Rhode Island, the Missouri DOT, the City of Chicago and The Illinois Tollway Authority all reported significant success in using wet process and/or terminal blend rubber-modified asphalt in large quantities over the last decade in colder climates [68]. The Illinois Tollway noted the FHWA requirement to use recycled materials whenever possible [49], but the Tollway also noted that there was little or no economic incentive to use wet process rubber in place of polymers in modified asphalt applications [68].

Minimal current national growth in demand for wet process and terminal blend rubberized asphalt appears to be driven by state-level mandates for rubber use. Experimentation in other state markets continues, but demand growth for the product appears to be flat. Applications across a range of pavement designs and use environments appear to be successful. State mandates/market incentives and/or assignment of some sort of premium for the reuse of rubber in pavements might boost production growth.

### 3.2. Dry Process

Crumb rubber-modified asphalt began to take root in the U.S. asphalt market in the early 2000s. Testing and commercialization of the dry mix process (the introduction of engineered crumb rubber at the producer’s site during the production of hot and warm-mix asphalt) was among those efforts. In the dry process, crumb rubber is added to the hot aggregates, similar to reclaimed asphalt pavement (RAP) at the plant, and then mixed with binder. Typically, fine-sized rubber particles (between 0.4 and 0.6 mm in diameter with an average particle size of 0.5 mm [4] are used. The rubber may be added on top of the mix or replace fine aggregates. Rubber content rates range from 5% to 20% of the virgin binder content.

Introduction of rubber using the dry process includes the use of engineered crumb rubber, designed to reduce mix stickiness, improve workability, increase the ease/speed of binder uptake into rubber and ease the introduction of rubber into the asphalt production process while producing a high quality and reliable asphalt mix. One of the most successful dry process efforts uses a metered pneumatic feeding system to inject fine, engineered crumb rubber into the mill during asphalt production, creating an asphalt rubber composite that performs as well as wet mix and polymer-modified asphalt. Depending on the performance criteria for the modified asphalt, these processes typically cost 15 to 50% less than wet process rubber-modified and polymer-modified asphalt binders. In general, the addition of 10% tire rubber by weight of the asphalt binder results in a two performance grade (PG) bump. However, the asphalt binder content in the mix should be increased by 0.2 to 0.4% for proper coating of fine particles in the mix and to offset the absorption of binder into the crumb rubber matrix.

The Georgia DOT was the first state to specify both the wet process and the dry process rubberized asphalt as an alternative to polymer-modified asphalt. The wet process has made no headway in Georgia, and polymer modification has lost market share. In Florida, a minimum level of terminal blend rubber use in asphalt was mandated by the state. Now that the state allows contractors to choose between polymer modification and terminally-blended rubber in modified asphalt projects, the use of terminally-blended rubber has stopped in Florida. Rubber has an important place to fill in the asphalt pavement industry. To facilitate the growth of rubber use in asphalt pavements, an economic incentive or state mandate for using rubber in asphalt pavements is required.

In addition to economic benefits, improvements in pavement performance have also been reported as a result of using the dry process CRM in asphalt mixes. Cao [69] indicated that the addition of scrap tires to asphalt mixes using the dry process improved asphalt mix performance. It was concluded that the amount of rubber added to the mix significantly affected the high temperature resistance of the mix to permanent deformation and low temperature cracking [69]. As the rubber particles are relatively large, this process is suggested for gap-graded mixes that provide space for large rubber particles. The rubber modification’s potential to improve fatigue resistance and ice control in pavements has been shown in different studies [70,71]. These benefits are due to the elastic behavior of rubber, which results in more flexibility of asphalt mixes. In addition, a research study conducted in Italy indicated that fatigue performance of dry-processed CRM mixes containing up to 3.5% crumb rubber by weight of the aggregates was broadly comparable to conventional mixes [72]. Visual inspection of rubberized porous European mix (PEM) indicated that rubberized PEM performed similarly to polymer-modified asphalt pavement sections in terms of rutting and cracking [73]. Field measurements confirmed that rubberized asphalt paving reduced traffic noise from light-duty vehicles [74]. Although there might be interaction between CRM and asphalt binder during mixing, storage, hauling, placement and compaction, the asphalt binder is not assumed as a modified binder in the dry process. This process is generally used for hot mix asphalt.

In another study, Hassan et al. [75] indicated that the critical design factors for designing dry-processed CRM mixes were: aggregate gradation, rubber gradation, binder content and air voids content. The following general guidelines for dry process CRM mixes were suggested by these researchers:

- Gap-graded or coarse, densely-graded aggregates are preferred;
- Same binder grade or higher penetration binder must be used compared to HMA;
- Higher binder content should be used compared to HMA (1–2%);
- Combination of coarse and fine rubber is desirable;
- Low design air voids content is critical (approximately 3%);
- A higher mixing temperature (compared to HMA) must be used;
- Rubber must be added to hot aggregate prior to adding the binder;
- 1 to 2 h of curing time is needed after mixing.

Different production and construction aspects of dry and wet processes are summarized in Table 3.

#### 3.2.1. Laboratory and Field Data on Effectiveness of Dry Process

Despite mixed performance data reported for the asphalt mixes containing dry process CRM, the dry process is known to show promising outcomes. After more than a decade of laboratory and field work, it is more widely accepted that the dry process works as well as the wet process and polymer-modified asphalt. The following sections present an overview of laboratory and field testing that led to the development of the dry process.

##### Published Lab Work

There has been a steady stream of work studying the addition of rubber to asphalt mixes. A majority of these works directly or indirectly focused on the dry mix processes. A summary of the more substantive laboratory efforts that helped shape the use of the dry process along with a discussion of the salient points from each effort is presented in this section.

Federal Highway Administration SuperPave^®^ Binder Modification Research: 

Under the direction of Terry Arnold and John D’Angelo [76], the U.S. Federal Highway Administration performed evaluations of rubber additions to various asphalt binders. The testing evaluations also included an industrial wax additive used to reduce the stickiness of the rubber-modified binders. Rubber was added in crumb form (ASTM-compliant 14 to 30 mesh material) at a 5% by weight of binder rate, both with and without the workability additive. The crumb rubber was mechanically blended with a shearing mixer at 160 °C for 30 min and was not cooked or otherwise digested at high temperatures for extended periods like terminally-blended materials. The true grade of the test binder (B6225 binder, TFHRC Lab ID) was measured at 67.0–25.3 °C. With or without the addition of the workability additive, the rubber-modified binder was graded as PG 70-22 with a true grade that ranged from 70.3–26.1 °C to 74.4–27.1 °C (with the workability additive). In general, the testing revealed that the addition of approximately 5% rubber by weight of the binder would raise the PG one grade. The addition of rubber could marginally decrease the low PG temperatures as well. The viscosity of the binders also increased with the addition of crumb rubber. This is possibly due to the asphalt binder’s light ends being absorbed by rubber.

Key Findings

- Addition of 5% crumb rubber will increase binder viscosity and raise the performance grade by approximately one level, depending on the type of the base binder used.
- The crumb rubber was not subjected to extended cooking and still had a beneficial effect on the modified binder.

Heiden Labor Vest Binder Tests of Crumb Rubber-Modified Binder.

A German asphalt consultant studied the effects of rubber and workability agents on asphalt binders [77]. Two binders were used in the evaluations: 50/70 and 70/100. These were approximately equivalent to SuperPave^®^ PG 64–22 and PG 76–22, respectively. The 50/70 binder was evaluated after 5%, 10% and 15% rubber additions, and the 70/100 binder was evaluated after 10% and 15% rubber additions. From these analyses, a 5% mix equated to 95% binder and 5% crumb rubber.

The modified binders were subjected to the following traditional binder analyses:

○ Needle penetration
○ Ring and ball softening point
○ Ductility
○ Elastic recovery
○ Deformation behavior (thermal stability) with a Dynamic Shear Rheometer (DSR)
○ Low-temperature behavior with a Bending Beam Rheometer (BBR)
○ Deformation work

The binder samples were mixed with crumb rubber of undisclosed particle size. Additionally, the binder, rubber and additive were combined in a paddle mixer (low shear) at 180 °C for 120 min before testing. The 50/70 binder mixes, containing 5%, 10% and 15% rubber, respectively, showed an increase in viscosity with more addition of rubber. All samples passed needle penetration and elastic recovery tests. Complex shear modulus, phase angle, m-value and stiffness results were all rated good to excellent, but m-values decreased as the ratio of rubber to binder increased. The 70/100 binders with 10% and 15% crumb rubber blends were also evaluated and showed similar results.

Key Findings

- Rubber additions enhance rutting resistance in direct relationship to the amounts of rubber added.
- The addition of increasing amounts of rubber appears to have a small negative effect on m-values and associated cracking resistance; as more rubber is added, it will bring the critical low (PG) temperatures slightly higher. However, it does not capture crack pinning potential as a result of using rubber as reported by Rath et al. [39].

Penn State NECEPT Binder and Mix Study 

The effects of the addition of crumb rubber to asphalt with an industrial wax product on both binder and mix performance was investigated in a study conducted by Solaimanian et al. [78]. A PG 58-28 binder from Wichita, KS was used with variable addition rates of crumb rubber with particle sizes of less than 1.397 mm (No. 14 sieve). The crumb rubber and modified crumb rubber were added to the binder at 150 °C and were subjected to shear mixing for 30 min, which was a fair simulation of the violent mixing and hold time associated with dry process for CRM asphalt hot-mix designs. Their work continued to build on the growing body of research associated with rubber additions to asphalt. It is important to note that rubber grain size and amount, binder quality, binder temperature and dwell time are among the key parameters which control the quality and performance of the final product. Their findings are summarized as follows:

Key Findings

- Binder viscosity increased by 75–80% with an addition of 10% crumb rubber, with particle sizes finer than 1.397 mm (passing a No. 14 sieve), as a fraction of binder in the mix design.
- For the selected binder, one-grade and three-grade increases in PG grades were observed with addition of 5% and 10% rubber, respectively.
- When compared to the PG 58-28 neat binder used in the study, rutting resistance of unaged and rolling thin film oven (RTFO)-aged binder blends increased exponentially with an increase in the amount of the rubber, up to 10% of binder content
- Fatigue testing of modified binders showed improvements in performance with an addition of 10% rubber with minimal cooking times and high shear mixing for both short-term and long-term aged binders.
- Addition of rubber to unaged binders resulted in a higher viscosity at 135°C, but they still easily met AASHTO standard (M320-2) for pumpability.
- Bending Beam Rheometer (BBR) tests showed marginal improvements in low temperature performance with the addition of crumb rubber with particles passing a No. 14 sieve. The m-values showed little or no change with the addition of rubber.
- Preliminary testing of mix designs suggested that using rubber in the mix was effective in reducing the susceptibility of asphalt mixes to rutting.

This study supported the positive effects of rubber-modified binder on asphalt mix performance. Additionally, it suggested that a significantly shorter cooking or digestion time when shear mixing was used did not negatively impact CRM asphalt pavement performance.

Rutgers University: Dry Process Evaluation 

Bennert et al. [79] compared the performance of an unmodified PG 64-22 binder with a polymer-modified PG 76-22 binder. The asphalt binder was modified with addition of 20% rubber using both wet process and dry process. Dynamic shear modulus (DSM) testing indicated that the polymer-modified binder was significantly more rut-resistant than the unmodified one. Additionally, all of the rubber-modified asphalt samples were marginally more rut-resistant than their polymer-modified counterparts. Permanent shear strain testing on a range of samples demonstrated that all of the polymer-modified binders performed significantly better than a standard PG 64-22 binder. Based on this study, the wet and dry mix rubbers performed marginally better than polymer-modified ones.

Key Findings

- Both wet and dry process CRM mixes performed at least as well as PMA, if not better.
- Dry process CRM mix performance was competitive with the wet process mix and better than PMA in all key test categories.
- The CRM pavements were more resistant to fatigue and moisture-induced damage than the PMA binders.
- Wet and dry CRM mixes accumulated approximately 18% less permanent shear strain at 5000 cycles compared to PMA.

NCAT Report

A follow-up study by NCAT [2] focused on answering three outstanding questions: (i) Does the process of recycling rubber (cryogenic versus ambient) impact the performance of crumb rubber in asphalt? (ii) Does the particle size distribution of crumb rubber impact the performance of rubber-modified asphalt? (iii) Do laboratory methods simulating dry process CRM asphalt show any loss in pavement performance compared to wet process asphalt modification?

Samples of rubber were taken from two tire recyclers that produced cryogenic- and ambient-recycled crumb rubber. Eleven different samples of rubber (different production processes and different particle size distributions) were collected and used on a single binder in order to create 13 different modified binders. Eleven of the samples included 10% crumb rubber modification and 2 out of the 11 were further modified to bring the CRM level up to 15%. The rubber-modified binder samples were prepared using short-term high-shear mixing. Each sample was evaluated using PG analysis, multiple-stress creep recovery (MSCR) and separation tube/softening point settling. Additionally, the source of the recycled rubber (auto versus truck tires) was tracked.

Crumb rubber particle sizes ranged from smaller than 1.4 mm (passing a No. 14 sieve) to smaller than 0.106 mm (passing a No. 140 sieve) from both cryogenic and ambient sources. Mean particle sizes ranged from 50 to 600 microns. The particles were substantively the same in chemical makeup, but surface areas exhibited significantly different values, ranging from 0.044 to 0.75 m^2^/g of crumb.

In the mix designs, a PG 70-22 binder (polymer-modified) was used as the base binder. Modification with 10% rubber produced a PG increase of one to two grades, and almost all samples were close to or over PG 82-22 grade. This was consistent with earlier observations that a 10% addition of rubber produced a two high-temperature PG bump. The mean high-temperature grade was 81.5 °C, with a 10% addition rate for crumb rubber. Low-temperature grading tests showed that an addition of 10% rubber to the binder followed by shear mixing produced rubber-modified binders that averaged a low PG temperature of −23.4 °C. This was approximately 0.6 °C higher than the rating of the continuous grade of the unmodified binder (−24 °C). This was consistent with the observation suggesting that greater unengineered crumb rubber additions will move both the high and low PG temperatures higher. This observation was also supported by the fact that both 15% crumb rubber-modified binders failed to meet a −22 °C specification. In general, the grinding method, temperature and particle sizes appeared to have no impact on either high or low temperature PG ratings.

MSCR testing of various mix designs demonstrated that almost all mixes qualified for traffic volumes in excess of 30 million equivalent single-axle loads (ESALs) for E-rated pavements. Separation tube/DSR analysis demonstrated evidence of significant separation or settling in all samples. Samples from the top and bottom of settling tube samples were subjected to a critical high-temperature evaluation and the mean variation between top and bottom critical high temperatures was found to be approximately 21 °C, with the higher critical temperatures found in the bottom samples. This suggested a mean separation of approximately 20% of the rubber additive; larger particles separated the fastest. Softening point analysis showed that all but one of the samples had a greater than 2 °C variance between upper and lower level-modified binder samples, thus reinforcing the risk of separation in CRM binders.

The final portion of the NCAT study focused on the effects of polymer and workability agents on the performance of CRM asphalt binders. The following three types of materials were evaluated: (i) straight CR was added at 10%; (ii) rubber-modified with a workability additive was added at 10%; and (iii) a proprietary rubber/polymer hybrid was added at 10%. The PG grades of all tested binders were similar. MSCR testing indicated that the workability additive marginally improved elasticity; however, softening point testing indicated that the same additive increased the rate and amounts of rubber separation in CRM binders.

Key Findings

- 10% and 15% addition of rubber produced close to a two-grade increase in high-temperature PG.
- Short cooking of binder/rubber blends and shear mixing had no apparent material impact on the quality of the binder compared to longer periods of digestion.
- Neither the type of rubber (truck versus auto tires), the type of grinding processes (cryogenic versus ambient) nor the particle size distribution had a significant impact on the performance of CRM binders.
- Addition of more CR to binders increased the critical low-temperature PG temperatures of the modified binders.
- Without properly engineered agitation systems, settling is an issue for wet-blended CRM binders.
- Use of workability agents or polymer/rubber hybrids did not appear to materially improve binder performance (PG, MSCR, Settling).

Illinois Tollway I-88 Ground Tire Rubber Test Sections 

Utilization of GTR in mixes used for construction of asphalt pavements in the state of Illinois has resulted in an improved resistance to rutting and cracking as well as environmental advantages [80]. Two different new GTR technologies—namely Elastiko 100 Engineered Crumb Rubber (ECR) and Evoflex Rubber-Modified Asphalt (RMA)—as well as terminally-blended GTR have been used by the Illinois Tollway in its projects. Evoflex RMA is an engineered workability enhancer engineered with GTR, SBS and other additives. The ECR technology is a dry process engineered GTR. The Illinois Tollway’s experience with terminally-blended GTR in the Chicagoland area with significant GTR mix placement volume has been successful and has resulted in field sections with good performance. In a study conducted by Buttlar and Rath [80] for the Illinois Tollway Authority, several mixes and field sections were evaluated and their resistance to rutting, and cracking was characterized.

Key Findings

- From several mixes tested, only one mix failed the required fracture energy failure criterion from conducting the disk-shaped compact test at −12 °C, and two mixes tested at −18 °C, an indicator of reasonable resistance to thermal cracking.
- Modeling showed that all mixes tested in the study should stay crack-free at low temperatures during their in-service life. All three GTR technologies and mixes characterized in the study were found to show promising performance against thermal cracking and rutting.
- Tested mixes showed high fracture energy and a reasonable creep and relaxation behavior, with no thermal cracking potential and superior excellent rutting resistance.
- The results showed that Elastiko and Evoflex had a similar effect on the embrittlement temperatures, while the Seneca GTR mixes showed the coolest embrittlement temperatures. AE testing on gyratory samples revealed that the use of a softer binder decreased the embrittlement temperature and the addition of recycled material increased it, as expected.

###### Field Work

A summary of the more substantive field efforts that helped shape the field use of dry process, along with a discussion of the salient points from each effort, are presented in this section.

Georgia Department of Transportation (GDOT) Evaluation of Dry Process Rubber Pavements Following Extended Field Service

An extensive investigation of multiple interstate highway projects using dry process rubberized asphalt in porous European mixes (PEM) and stone mastic asphalt (SMA) mix designs was conducted by Shen et al. [54]. These designs were evaluated after three and five years of service. Each project had an adjacent control lane constructed with the same mix designs using polymer-modified asphalt binder in place of dry process rubberized asphalt. The authors used a combination of field performance evaluations and core analysis along with two extensive laboratory analyses of mix performance. Based on the field investigations and evaluation of cores, the authors found no material evidence of any significant difference between PMA and dry process rubber performance. The authors also evaluated the performances of wet process and dry process CRM asphalt and rubber- and polymer-modified pavements placed side-by-side. No material differences were found in field performance between the different modification processes. Although the authors did not update their findings in the most recent report, the very first interstate section, using dry process rubberized asphalt with PMA as a control, showed similar life-cycle performance after 14 years of service on I-75. Both pavements were removed in 2020, at which time both were in a similar condition.

The laboratory analyses were also far-reaching, but several key conclusions were noted, as follows.

Key Findings

- Rut resistances of both dry process rubber and polymer-modified binders were found to be similar.
- Within 30–60 min of mix production, dry process asphalt mimicked the performance of wet process asphalt across a wide range of binder and mix tests.
- Dry process rubber-modified binders exhibited strong similarities to wet process and PMA binders in their respective master curves.
- The mixing associated with the turbulence and abrasion found during asphalt production acted to accelerate rates of light end segregation with the dry process.
- The researchers concluded that the dry process for rubberized asphalt mix designs could be used effectively in the field.

Illinois Tollway Lab and Field Evaluation 

The Illinois Tollway (Tollway) began working with rubberized asphalt over the last decade [80]. The agency has used hundreds of thousands of tons of rubberized asphalt in their system, and that asphalt is performing at or beyond design expectations. Tollway has been using both wet and dry asphalt modifications and has devised its own system of mix testing in laboratories to predict the expected field performance of various asphalt mix designs [80]. Based on Tollway’s suggestions, rubberized asphalt should be competitive with PMA in the field. In a study conducted by Buttlar and Rath [80] for Tollway important findings were reported a summary of which is shown here.

Key Findings

- Extensive field testing of CRM asphalt mixes and similar mix designs with PMA was conducted. The data indicated that the field performance and expected life of rubberized pavements (GTR sections), as measured in the field using a pavement condition rating system, had a total expected service life of 17 to 23 years. However, sections constructed using PMA mixes had a total expected service life of 12 to 18 years. At a minimum, the field performance of the tested mixes with GTR appear to be comparable to those constructed using mixes containing PMA.

#### 3.2.2. Summary of Lab and Field Work

The findings from the aforementioned laboratory and field studies can be summarized as follows:

○ Crumb rubber additions of approximately 5% and 10% produced one and two PG grade increases in asphalt binders, respectively.
○ Rubber size, type, and grinding process affected—but did not significantly impact—binder or mix modification.
○ Use of CRM binders was found to effectively improve rutting resistance. More rubber resulted in a stiffer mix—but more rubber alone also elevated critical low temperatures and led to premature cracking, which is not desirable.
○ Crumb rubber cooking time in binder beyond 30 to 60 min did not significantly improve binder performance.
○ With proper mixing, low- and no-cook (dry process) CRM asphalt seemed to perform as well as terminal-blend CRM asphalt, both in the lab and in the field.
○ Wet and dry process CRM asphalt performed as well or better than PMA with respect to fatigue, permanent strain accumulation and resistance to moisture-induced damage.
○ Separation of rubber and binder in terminal blends was a technical challenge.

#### 3.2.3. Production and Construction Aspects of Dry Process CRM Asphalt

Various forms of CRM asphalt pavements using dry process have been used in the field for more than a decade, with total pavement placements exceeding four million tons. Projects have ranged from initial efforts in parking lots and on municipal streets to multiple projects on state and interstate highways. At present, dry process CRM Asphalt is in use on more than 5000 lane-miles of pavement on more than one hundred significant projects located all over the U.S. in heavy-use transportation interstate corridors [81].

The Georgia Department of Transportation (GDOT) engaged independent researchers to evaluate the last decade’s worth of dry process pavements in the state, with particular attention paid to interstate highway projects on I-20 and I-75. Test sections of up to 17 miles in length were placed in 2008–2012, and each test section had a polymer-modified control section of similar mix design included in the project design. Through 2020, dry process and control test sections exhibited no significant difference in either rutting or cracking performance. As of the end of 2020, Georgia will have more than 1.5 million tons of dry process asphalt in service. The process specified in Georgia is permitted in Louisiana, and the process is under various stages of review in a number of other states [81].

In view of the outcomes of the cited literature, the following observations were made associated with dry process CRM asphalt in the field:

- Engineered rubber infeed rates appeared to have a narrow range of variation: ±2% according to conveyancing machine manufacturers and field measurement. State specifications limit this variation to ±5%.
- There is no record of any project showing material areal variations in pavement performance.
- All plant-produced dry process CRM asphalt materials met applicable production specifications.
- There were no problems with basic operation of metered feeding systems and tracking of inputs.
- All dry process CRM asphalts exhibited excellent workability, good to excellent compaction, good to excellent minimum compaction temperatures, low field emissions and minimal stickiness during handling—even for rubber asphalt applications. This is true for breakdown temperatures as low as 113 °C.
- With proper workmanship, placed pavements using 7–10% rubber as a fraction of binder have shown performance comparable to, if not better than, polymer-modified PG 64-22 binders with a true grade of PG 76-22.
- Comparative evaluations between dry mix CRM asphalts and polymer-modified asphalts (3 lbs. of SBS per 100 lbs. of binder, as low as 6.4 lbs. of rubber per 100 lbs. of binder) and wet mix rubberized asphalt showed no difference in performance in the field, including heavily-travelled interstate highways over periods as long as 1.5 decades.
- Within limits, the size, type, grain size distribution (within a modestly narrow range) and processing method for crumb rubber do not seem to materially impact the quality and performance of the paved surfaces.
- The method for rubber introduction into asphalt (wet or dry) does not have a material impact on the quality or the performance of the paved surfaces.
- The amount of rubber added, temperature, dwell time, ratio of binder to rubber additions and the engineering of the rubber appear to be the most important variables in regard to pavement performance.

#### 3.2.4. Performance Comparison between Wet and Dry Process CRM Mixes

Semi-circular bend (SCB) tests conducted on gap-graded CRM asphalt mixes indicated that asphalt mixes produced by wet and dry processes resulted in mixes with better fatigue performance [82] compared with unmodified mixes. Similar results were obtained by conducting flexural bending beam fatigue tests [83]. Losa et al. [84] investigated the permanent deformation and fatigue cracking resistance of gap-graded CRM mixes produced using both wet and dry processes. Similar results were obtained for both wet and dry process mixes based on indirect tensile strength tests (IDT). However, the resilient modulus (Mr) test data indicated that wet process mixes had higher Mr values compared to dry process mixes. The indirect tensile fatigue test results indicated that gap-graded mixes prepared using the dry process could be superior to wet process mixes in terms of fatigue cracking resistance. In another study, Kim et al. [85] conducted research on CRM asphalt mixes in Korea. In that study, asphalt mixes produced using dry and wet processes with CR contents of 8%, 10%, and 12% were investigated. The laboratory results indicated that fatigue performance of the CRM mixes at 2 °C showed the most significant improvement. It was concluded that wet process CRM mixes were stronger than dry mixes in terms of high temperature deformation resistance and tensile strength at ambient temperatures. It was shown that the moisture resistance of both dry and wet processed mixes was low after freezing-and-thawing cycles. The addition of hydrated lime was found to improve the resistance of the CRM mixes to moisture damage. Moreno-Navarro et al. [86] investigated the fatigue cracking potential of four different mixes with the same aggregate gradation but different binder types. It was concluded that the fatigue lives of mixes containing CR were considerably higher than those of conventional HMA. It was also shown that cracks in dry-processed mixes were thinner and lesser than those in other mixes.

Although dry process has potential for use in recycling high amounts of scrap tires in asphalt mixes, a lack of standards for this method has limited its use in the asphalt pavement industry [75]. Most of the inconsistencies were observed in laboratory studies due to a significant difference between lab-produced and plant-produced mixes or as a result of different mixing procedures and compaction efforts. However, there are many studies which have shown the superior performance of dry process CRM mixes. Therefore, widely accepted mixing and compaction methods that can represent both asphalt plant and field conditions are needed for laboratory testing of CRM mixes.

Summaries of the laboratory and field performance aspects of dry and wet processes are presented in Table 4 and Table 5, respectively.

#### 3.2.5. Experience of State DOTs with GTR Applications and Field Operations

The literature review indicated that there are a variety of applications for CRM materials throughout the United States. In this section, the experiences of different DOTs with CRM asphalt is discussed.

##### Florida

The Florida Department of Transportation (FDOT) is one of the agencies that has conducted extensive research and field experiments on CRM pavements. In a 1996 study, FDOT investigated the effect of different grinding processes on the asphalt rubber properties. They found that GTR with greater surface areas and more irregularly-shaped particles produced asphalt binders with higher viscosities. It was also concluded that asphalt binders with GTR produced using a cryogenic grinding technique had the most settlement and least drain-down resistance [50]. Three demonstration projects conducted by FDOT in 1989 and 1990 evaluated the constructability and short-term performance of asphalt rubber pavements with various amounts of GTR in a typical production project. The first two projects tested the stability and constructability of mix designs. Each of these projects used different binder contents, ranging from 3% to 17% depending on the project objectives. The third project explored the sensitivity of dense-graded and open-graded mix properties to changes in CR particle size and binder content.

Three test sections and a control section were included in the first project. Three GTR-modified binders, containing 3%, 5%, and 10% CR by total weight of binder, respectively, were used in this study. All of the mix designs were developed using Florida DOT’s Marshall mix design procedure. The binder content of 7% was selected for the control section, while the binder contents of the sections with 3%, 5% and 10% CR were 7.22, 7.37 and 8.25%, respectively. During construction and placement of the mixes, problems were observed, such as occurrence of mix pickup with the rollers and tenderness of the section with 10% CRM. Experimental tests on plant-produced materials indicated that all the sections except that containing 10% CRM had similar Marshall stabilities with their design values. The stability value of the section with 10% CRM was half that of the design value. It was hypothesized that the reduced stability was due to higher binder content and lower fine particles [87].

Four test sections and a control section were included in the second project. Four GTR-modified binders with 5%, 10%, 15%, and 17% CR by total weight of binder were used in the study. The total binder content of 6.3% was selected for the control section and the binder contents of the sections with 5%, 10%, 15% and 17% CRM were 7.16, 8.11, 9.18 and 10%, respectively. The construction process indicated that the mix with 10% CR had the best constructability [87].

In the third project conducted by FDOT, four different sections were included in the construction. The results of this study indicated that dense-graded mix properties were more sensitive to changes in CR particle size and binder content than those of open-graded mixes. This sensitivity was attributed to the lower amount of void space available in dense-graded mixes. Based on the results of this project, FDOT drafted specifications for using crumb rubber in asphalt pavement surface course, validated by other researchers [88]. These specifications are still in use. For the dense-graded and open-graded surface courses, the CR amount was limited to 5% and 12% by weight of asphalt cement, respectively. The maximum size of ground rubber was set at 300 mm (no. 50 sieve) for dense-graded mixes and 600 mm (no. 30 sieve) for open-graded mixes [89].

In 1999, a study was conducted on the three above-mentioned projects to evaluate their performance. The major finding of this 10-year study was that the application of scrap tires in asphalt pavements using the wet process improved the crack resistance of surface mixtures. The test sections constructed using CRM mixes showed about 1–6% cracked areas, depending on the CR amount. However, the test sections constructed using mixes containing virgin binder or dry-mixed sections showed about 30% cracked areas. The CR amounts ranging between 10% and 15% were suggested as effective optimum rubber contents in this study [88]. According to the Florida Department of Environmental Protection (FDEP), Florida is the only state that uses modified rubber asphalt in the friction course of all state-maintained roads [90].

FDOT started the use of CR asphalt mixes in interlayers and seal coats about 45 years ago. FDOT allowed the use of CRM in surface treatments and interlayers based on the finding of a project conducted in 1980 [91]. SAMI binders in Florida include 20% CR by weight of asphalt in order to achieve a high viscosity material. In the most recent revision of FDOT asphalt mixture guidelines, the use of scrap tires in asphalt friction courses and membrane layers was approved [92].

##### Arizona

Arizona Department of Transportation (ADOT) has more than 45 years of experience incorporating asphalt rubber materials in the construction and rehabilitation of pavements. Rather than using crumb rubber in asphalt paving mixes, ADOT used GTR-modified binders in chip seals as stress-absorbing membranes (SAMs) and SAMIs. In a 1994 study, ADOT evaluated the service life of the various CRM treatment materials using the data available in the ADOT pavement management system data base. They concluded that the average service lives of SAMs were 6.4, 10.3, and 8.9 years, while the SAMIs average service lives were 10.7, 9.5, and 10.7 for interstate highways, state routes, and U.S. routes, respectively [93].

Although Arizona has a wide range of climate zones from hot (Yuma, Bullhead City) to cold (Flagstaff, Grand Canyon), there have been many successful pavement constructions using GTR-modified materials throughout the state [8,93,94,95,96,97].

ADOT designed and constructed a large-scale project in 1990 to evaluate the effects of asphalt rubber on reflective cracking occurrence in thin overlays. The project was located in Flagstaff, AZ on Interstate 40. The overlay project was constructed on a badly-cracked concrete pavement which was originally built in 1969, with a thickness of 8 inches and total width of 38 feet. The asphalt rubber for the project was produced with 20% GTR using the wet process. No other additives or modifiers were used in this project. It was reported that the performance of the asphalt rubber overlay was beyond the original expectation [96]. It was indicated that, after nine years of service, the overlay was still virtually crack-free, with no rutting. The results of this project led to significant increases in asphalt rubber application throughout Arizona.

Over 2000 miles of asphalt pavements containing GTR were built in Arizona between 1990 and 2000. ADOT tracked all the asphalt rubber overlay projects in the state and reported that the cracking percentage in asphalt rubber overlays was significantly lower than that in conventional overlays without any rubber. There have been many gap-graded CRM mixes placed in the State of Arizona. Kaloush et al. [98] conducted flexural fatigue tests on field specimens extracted from the gap-graded Arizona pavements and showed that crumb rubber modification resulted in higher pavement fatigue life. In a more recent study, point bending tests were conducted on Arizona mixes, indicating that CRM mixes had longer fatigue lives compared to traditional HMAs. These results were consistent with field observations over a 16-year period [99]. An Arizona Transportation Research Center study also reported noise reduction as an additional benefit of using CRM asphalt in pavement construction [100]. It was indicated that an asphalt rubber open-graded friction course (AR-ACFC) reduced noise by 5.7 decibels. Studies by ADOT on the use of scrap tires in pavements revealed that the thickness of CRM pavements was half that of conventional pavements. Furthermore, the cracking percentage in CRM mixes was approximately one-fourth that of conventional mixes over a similar period of time, making them an appropriate choice for the state [101]. In the state of Arizona, all high-volume highways have been surfaced with asphalt rubber open-graded friction course [47].

##### California

The California Department of Transportation (Caltrans) has been using scrap tires in chip seals since the 1970s and began using them in rubberized HMA in the 1980s [102]. Approximately 31% of all HMA mixes placed in California by the end of 2010 were rubberized HMA [102]. As mentioned earlier, the first Caltrans dry process CRM HMA pavement was constructed in 1978. The first Caltrans rubber asphalt concrete (RAC) pavement to use wet process was constructed in 1980. Caltrans’ successful experience with CRM asphalt changed their approach to the use of high viscosity CRM binders. The constructed pavements were monitored over time and the overall performances of the CRM mixes were rated excellent by Caltrans [103]. Caltrans built more RAC projects and continued studying their performances. It was clear by 1987 that thin RAC pavements performed better than thicker, conventional dense-graded asphalt concrete (DGAC).

Caltrans’ engineers reviewed the performance of over 100 RAC projects in California and 41 Arizona DOT projects. It was concluded that the performance of RAC mixes was excellent when properly designed and constructed. A very important finding of the study was that the progress of distresses in RAC pavements was much slower than that of structurally equivalent DGAC pavements. A total of 210 RAC projects were constructed by Caltrans by mid-2001. Caltrans also included SAMIs in the pavement construction for a project in Ravendale, CA. The results of this study significantly changed Caltrans’ approach to the use of CRM materials. It should be noted that the vast majority of Caltrans’ rubber-modified pavements used the wet process.

##### New Jersey

The New Jersey Department of Transportation (NJDOT) conducted a study on seven experimental field projects to evaluate the use of wet and dry processes, including control sections, constructed from 1991 through 1994. The results of this study indicated that the wet process DGAC mixes performed similarly to DGAC mixes without GTR. The asphalt plant emissions tests were conducted on three wet process CRM mixes containing 10%, 15% and 18% crumb rubber by asphalt binder weight. It was reported that emissions—namely, total hydrocarbons, carbon monoxide, and particulate emissions—for two aforementioned mixes were within the emissions limits set by the state of New Jersey while the third wet process was found to have some emissions above the limit. A proprietary gap-graded dry process CRM mix was also produced, and its total hydrocarbon emission was found to be above the emissions limit [104].

##### New Mexico

The New Mexico Department of Transportation’s (NMDOT) first experience with dry process CRM HMA was in 1984. The performance of the pavement was monitored for nine months and it was reported that the pavement structure performed well during winter months. However, during the hot weather, the pavement lost structural capacity and failed. As stated by the report, the pavement “literally came apart.” In 1985, NMDOT constructed the first wet process CRM pavement. Within the first year of service, the pavement surface showed excessive premature cracking. After these two unsuccessful projects, NMDOT stopped using crumb rubber in asphalt pavements for ten years [105]. In 1994, six rubberized, open-graded friction course (ROGFC) projects were constructed by NMDOT. Monitoring the performances of the sections indicated that the mixes performed better than conventional open-graded friction course (OGFC) pavements in New Mexico. It was also reported that the cost of ROGFC was 33% higher than that of conventional OGFC [105].

##### Texas

The Texas Department of Transportation (TxDOT) has 40 years of experience in utilizing asphalt rubber in construction and rehabilitation of pavements. In Texas, CR has been used in four different types of pavement constructions: chip seal coat, underseal, HMA and porous friction course (PFC) [106]. In a study conducted in 1982, researchers evaluated the performance of nearly 800 miles of Texas seal coat and underseal projects constructed from 1976 to 1981. The results indicated that using asphalt rubber binder in seal coat construction reduced alligator cracks and raveling compared to seal coat coats constructed using conventional HMA [107]. In a latter study conducted in 2002, it was concluded that asphalt rubber chip seals were a good treatment option for Texas pavements [108]. Pavement evaluation results indicated that CRM HMA projects had significantly better cracking resistance than conventional HMA. Good rutting resistance was also reported from CRM HMA projects [109]. In a study conducted for TxDOT in 2001, it was stated that “all asphalt rubber Porous Friction Course (PFC) projects are exhibiting excellent performance properties. Resistance to cracking and raveling in asphalt rubber PFC is particularly impressive. From cost and benefits standpoint, PFC represents the best application for asphalt rubber” [109].

##### Oregon

Seventeen test sections were constructed by the Oregon Department of Transportation (ODOT) from 1985 to 1994 throughout the state. These sections were evaluated through visual condition ratings (based on ODOT’s modified SHRP method) and ride values as measured by a South Dakota-type profilometer. The results indicated that performance of the dense-graded wet process and dry process CRM mixes was noticeably worse than the control sections. However, the open-graded mix with 12% CRM passing a 180 µm sieve (No. 80 sieve) performed slightly better than the control section. No construction issues were encountered with gap-graded dry process mixes, but raveling occurred shortly after construction. It was concluded that, among the tested sections, the dry process mixes exhibited the worst performance. It was also indicated that higher temperatures were needed in field operations for mixes with high-viscosity CRM binders compared to unmodified control mixes [110]. It should be noted that many studies conducted more than 10–15 years ago do not cover many changes in dry process over time and may include PlusRide pavements, which are not used in the modern dry process.

##### Alaska

Dry CRM process was used in several projects by the Alaska Department of Transportation and Public Facilities (AKDOT&PF). In some cases, good performance was reported for test sections in terms of resisting low temperatures, fatigue cracking and improving ice control characteristics. Overall, no significant differences were found between the performance mixes produced using dry process and control mixes [111,112].

From the foregoing discussions, it is evident that the departments of transportation have different experiences regarding the use of GTR in asphalt mixes. Table 6 summarizes the present specifications for terminal-blended CRM binders (with maximum viscosity of 1.5 pa.sec) in Arizona, Florida, and Texas. Texas DOT requires complete digestion of CRM. Table 7 summarizes the existing specifications for on-site-blended CRM binders (with minimum viscosity of 1.5 Pa-sec) in Arizona, Florida, California, and Texas.

## 4. State DOT Survey on Use of GTR in Asphalt Mixes

In order to promote successful use of GTR, it is imperative to develop specifications for utilizing rubberized asphalt by collecting information, common practices and specifications from state DOTs. Therefore, a survey of construction specifications used by different DOTs which allow the use of GTR in asphalt was conducted as a part of this study in 2016. Since DOT practices are generally not available in the open literature, this survey was found to be an effective tool for gathering data on current practices, including the methods, special provisions and specifications associated with the use of GTR in asphalt pavement by DOTs. The survey questionnaire was prepared in close collaboration with ODOT and the Oklahoma Department of Environmental Quality (ODEQ). The survey was conducted through an online data collection website to maximize the efficiency and productivity of the data collection process. The survey questionnaire was distributed among different DOTs with the help of ODOT Materials & Research Division. Based on the responses received, 37 state DOTs and agencies from the United States and 1 Canadian transportation authority responded to the survey. Overall, 74% of state DOTs participated in the survey (Table 8).

Based on the responses received, at the time of the survey, more than half of the participating DOTs (54%) allowed the use of GTR in asphalt mixes. States which did not allow the use of GTR in their mixes noted some technical reasons. Table 9 reflects the reasons cited by DOTs for not using GTR in HMA. As shown in Table 9, of the state DOTs which did not allow the use of GTR in asphalt mixes, 61% cited the higher cost of GTR in asphalt mixes as the main reason for not using it. Forty four percent (44%) of the DOTs surveyed mentioned their concern over the performance of asphalt mixes containing GTR. The performance concerns consisted of premature reflective cracks and concerns over blending quality and settlement in the tanks. However, a number of states (AK, PA, and WI) noted improved performance of asphalt pavements as a result of using GTR in HMA. Thirty nine percent (39%) of the states that banned the use of GTR in HMA cited unsuccessful experiences using GTR in HMA in the past as their main reason for the bans. Lack of sufficient incentives to recycling scrap tires in asphalt roads was identified as a reason for 33% of the states which did not use GTR in HMA. Lack of performance data and crumb rubber producers were stated reasons for 28% and 22% of the states which did not use GTR in asphalt mixes, respectively.

Table 10 reflects the reasons cited by DOTs for using GTR in HMA. In total, 67% of the DOTs which allowed using GTR in asphalt pavement construction mentioned improved performance of the CRM mixes compared to conventional HMA as their main reason for using GTR in HMA. The performance benefits mentioned by DOTs as a result of using GTR included better thermal cracking resistance, better durability when used in OGFC pavements, successful use in hot rubber chip seal, cost-effectiveness as an alternative to polymer modification, satisfactory performance compared to PMA mixes, improved resistance to moisture-induced damage, considerable noise reduction, superior rut and crack resistance and better overall durability. Additionally, 25% of the agencies allowing the use of GTR in asphalt mixes mentioned the cost-effectiveness of CRM mixes compared to other options such as polymer modification as a reason for using GTR in HMA. Moreover, 21% of the agencies using GTR in HMA identified the incentives offered for using scrap tires in pavement as an important reason for their agencies’ use of GTR. Other reasons for the use of GTR in HMA—mentioned by 50% of the agencies—included environmental benefits and incentives offered by local departments of health to offset the costs of GTR-modified binders.

Table 11 reflects different GTR applications by DOTs. It was found that 87% of the DOTs/agencies allowing the use of GTR in asphalt mixes did so in HMA and 57% used it in warm-mix asphalt (WMA). Additionally, it was reported that, while 56% of DOTs used GTR in structural overlays, 52% of them used it in nonstructural thin-lift overlays. Moreover, 48%, 30% and 22% of participating DOTs, respectively, used GTR in mill-and-fill operations, chip seals and fog seal construction. Finally, 26% of the agencies which allowed the use of GTR in pavement used it for other applications such as dense and OGFC, crack sealant and for modifying asphalt binders to raise their PG grade.

Table 12 presents the applications of GTR in asphalt mixes with respect to the type of projects in which they were utilized. It was observed that 78%, 74% and 39% of states allowing use of GTR in their mixes used it in state highways, interstate highways and city roads, respectively. Additionally, 30% of the participating DOTs noted other types of projects. These applications included projects with a traffic level below 10 million ESALs, state highways with high rutting risks, major state routes with significant truck traffic (approximately 4000 ADTT or higher) and state routes with frequent stop-and-go traffic or significantly slow traffic on a steep grade.

Table 13 reflects the types of processes used by state DOTs in order to incorporate GTR or CR in asphalt mixes. According to Table 13, only 14% of the states which allow the use of GTR in asphalt mixes have used the dry process. However, 77% and 55% of the states have used the wet process, blended terminally and in the field, respectively. Although a majority of the states have used the wet process, some states have started investigating the benefits of using dry process in lieu of the wet process and reported success (e.g., MO).

Table 14 reflects the availability of guidelines, technical specifications or special provisions to states which use GTR in asphalt mixes. From Table 14, it can be observed that 86% of the states which use GTR in asphalt mixes follow specific guidelines for this purpose.

Table 15 shows technical considerations for asphalt mixes containing GTR recommended by DOTs participating in the survey. As shown, 50% of DOTs allowing the use of GTR in asphalt mixes require changing the mixing temperature when GTR is used. While a number of states (e.g., AZ, CA, ME, and NE) require mixing temperatures to be above 149 °C, some (e.g., NH, and NJ) require temperatures to be maintained below 149 °C in order to control odors. Additionally, 50% of states require a modification made to binder grade when GTR is used. While some states require the final product to meet PG 76-22 binder specifications, others recommend using a lower grade base binder to compensate for two (2) to three (3) PG grade increases as a result of using GTR in HMA mixes. Furthermore, 15% of DOTs require a modification in compaction effort when GTR is used. Moreover, other requirements, such as the use of WMA additives, additional binder viscosity testing and PG grading conducted by contractors are recommended by 30% of the DOTs which allow using GTR in asphalt mixes.

Table 16 and Table 17 reflect the criteria required by DOTs for the use of GTR in surface course and intermediate/base course mixes, respectively. Based on the responses received, while almost all of the DOTs using GTR in asphalt mixes try to maximize its use, most of them do not have maximum allowable GTR requirements. However, a number of agencies have more specific criteria in this regard. For example, the Nebraska Department of Roads (NDOR) typically uses 10% GTR by the weight of binder and has four separate specifications for incorporating GTR in mixes; namely, dry, wet terminal, wet plant and one to meet AASHTO M320 requirements. The New Hampshire Department of Transportation (NHDOT) typically recommends using 18% GTR by the weight of binder and suggests a viscosity test be conducted on the blended binder. Arizona DOT does not specify a maximum allowed GTR in the mix but requires a minimum of 20% GTR by weight of asphalt binder. Arizona DOT also runs rotational viscosity, softening point, penetration, and resilience tests on the crumb rubber asphalt. New Hampshire and South Carolina DOTs require a minimum GTR amount of 15% and 7% by the weight of asphalt binder, respectively. Georgia DOT does not specify a maximum amount for GTR; however, 8%–10% GTR by the weight of binder is typically used. A workability additive is required when GTR is used in a mix. Other standard performance test requirements, e.g., APA-rutting susceptibility, moisture susceptibility, and permeability should also be met by GTR-modified mixes. Louisiana Department of Transportation & Development (LaDOTD) typically uses 10% GTR by weight of asphalt binder and requires blended binder, wheel tracking and semicircular bend tests to be conducted on mixes. Although Wisconsin DOT does not have a limit for using GTR in mixes, it requires testing asphalt mixes for their susceptibility to low-temperature cracking. Caltrans has more specific requirements for using GTR in HMA. According to Caltrans, no more than 22% GTR by weight of asphalt binder is permitted in wet process. However, no upper limit for using GTR in HMA is specified in terminal blending processes. In addition, PG tests are required in terminal blending processes. Other requirements for asphalt binders set by Caltrans when wet process is used include viscosity, resilience, rebound and softening point tests. Maine DOT requires using a minimum of 15% GTR by weight of binder but does not specify a maximum amount.

Table 18 shows the major tests conducted by DOTs on asphalt mixes containing GTR. As shown in Table 14, 71% of DOTs using GTR in their mixes conduct rut tests (i.e., Hamburg wheel tracking and APA rut test) and moisture-induced damage tests (Hamburg wheel tracking and tensile strength ratio test). Only 18% of the DOTs indicated that they conducted fatigue test on mixes containing GTR. Sixty five percent (65%) of DOTs conduct permeability, semicircular bend (Louisiana method) and abrasion loss-of-mix tests (Cantabro test). Other tests conducted by 12% of DOTs include dynamic modulus, flow number and flow time tests.

## 5. Summary

The major outcomes of this study are listed in different categories, as follows.

### 5.1. General Mix Performance

1. Researchers reported enhanced mix performance resulting from utilization of scrap tire rubber in asphalt. The reported benefits include improved rutting resistance, thermal reflective crack resistance, resistance to fatigue cracking, reduction in maintenance costs, smooth ride, good skid resistance and noise reduction [5,7,8,9,10,12,54,79].
2. CRM binders have been shown to improve rutting resistance. More rubber results in stiffer pavements, but can also slightly elevate critical low temperatures. More rubber increases cracking resistance due to crack pinning and deflection.

### 5.2. Wet Process vs. Dry Process

1. Several studies reported that acceptable performance in an asphalt mix was achievable as a result of using both wet process CRM [88,113,114,115,116,117,118,119] (and dry process CRM [2,54,79,116,117] asphalt mixes.
2. Wet process crumb rubber cook time in binder beyond 30 to 60 min was not shown to materially improve binder performance.
3. With proper mixing, low- and no-cook CRM asphalt seemed to perform as well as terminally-blended CRM asphalt, both in the lab and in the field.
4. Both wet process CRM asphalt and dry process CRM asphalt, if properly engineered, designed and produced, have been shown to perform as well or better than PMA (better fatigue and moisture resistance, lower permanent strain accumulation).
5. Using dry process rubber could mitigate the risk of separation of rubber from asphalt binder that is associated with terminal blends.

### 5.3. Gap-Graded vs. Dense-Graded

Overall, the performances of gap-graded CRM when coarse CRM particles were used were found to be consistent. Gap-gradation provided sufficient space to use higher CRM contents and larger CRM particles (up to 2 mm) in comparison with dense-graded mixes. Due to the low void space in the aggregate structure of dense-graded mixes, they accommodated fine CRM particles (passing 300 µm sieve size or finer). Properly designed dense-graded CRM mixes performed similar to conventional DGAC [9,96].

### 5.4. SAMI Mixes

Evaluation and monitoring of the paved roads in Arizona and Florida indicated that application of CRM-modified SAMIs improved the overall pavement performance.

### 5.5. PG of CRM Binder

Crumb rubber additions of approximately 5% and 10% produced PG grade increases of one or two levels in asphalt binders, respectively.

### 5.6. GTR Size, Type and Grounding Technique

Rubber size, type, and grinding process affected but did not materially impact binder or mix modification. 

### 5.7. Major Outcomes of the DOT Survey

1. Based on the responses received, more than half of the participating DOTs (54%) allowed at least some use of GTR in their asphalt mixes.
2. The main reasons for not allowing the use of GTR in mixes were higher cost of using GTR in wet process (54%) and concerns over the performance of asphalt mixes containing GTR (44%). These concerns included premature reflective cracks, blending quality and settlement in the tanks. Other cited reasons for not using GTR in mixes included unsuccessful experiences using GTR in HMA in the past (39%), lack of sufficient incentives to use GTR in asphalt/cost (33%), lack of performance data (28%) and lack of crumb rubber producers in the state (22%).
3. The main reason cited by state DOTs for allowing the use of GTR in mixes were improved performance of CRM mixes compared to conventional HMA (67%). The performance benefits of GTR mentioned by DOTs included better thermal cracking resistance, better durability when used in OGFC pavements, successful use in hot rubber chip seal, cost-effectiveness as an alternative to polymer modification, satisfactory performance compared to polymer-modified asphalt mixes, improved resistance to moisture-induced damage, considerable noise reduction, superior rut and crack resistance and better overall durability. The cost-effectiveness of CRM mixes compared to other options (25%) and the incentives offered for using scrap tires in pavement (21%) were other reasons cited for using GTR in HMA. Other reasons for use of GTR in HMA mentioned by agencies (50%) included environmental benefits and incentives offered by the local environmental or health departments to offset the higher cost of GTR-modified binders (wet process).
4. Dry process was used to incorporate GTR in mixes by only 15% of responding states at the time the survey was conducted. In recent years, this ratio has increased to at least 25%.
5. Of the states allowing the use of GTR in their mixes, 86% followed specific guidelines for this purpose.

## Figures and Tables

**Table 1:** Key findings of selected studies conducted on asphalt binders and mixes.

Key Findings	Studies
Rubber-related enhancement in asphalt binder performance was observed compared to neat binder. Improved high-temperature PG when rubber was used Improved resistance to fatigue cracking when rubber was used Improved binder elasticity when rubber was used Improved aging characteristics when rubber was used Lower temperature susceptibility when rubber was used Higher GTR-binder interaction time (4–8 h) led to higher elasticity Higher moisture-induced damage potential in binder-aggregates was observed when rubber was used Storage stability of CRM binders is an issue Aliphatic components released from crumb rubber during degradation CR particles did not fully dissolve in asphalt binders regardless of their size	[6,7,8,9,10,11,12,13,31,32,34,35,36,37,38]
Rubber-modified asphalt pavements performed better than, or at least as well as, standard HMA pavements Improved resistance of mix to fatigue cracking when rubber was used Improved resistance to rutting when rubber was used Reduction in crack propagation rate when rubber was used Tire rubber can be successfully used in open-graded mixes and surface sealants 8-year field data of dry process showed an acceptable performance A higher raveling resistance was achieved when rubber was used While both dry and wet process CRM asphalt mixes both have shown adequate performance (rutting and fatigue), use of dry process does not need major modifications made to asphalt plants.	[4,5,11,18,33,39,40,41,42,43,44,45,46,47,48]

**Table 2:** Wet process, on-site vs. terminal blending.

Method	GTR Size	Rubber Amount	Mix Gradation Type
On-site Blending	1.4–2.0 mm	15 to 22%	Gap-graded or Open-graded
Terminal Blending	<0.6 mm	5 to 10%	Dense-graded

**Table 3:** Comparison of different production and construction aspects of dry and wet processes.

	Dry Process	Wet Process
*Production Process*	Crumb rubber is added to the hot aggregates similar to RAP at the plant and then mixed with binder The rubber may be added on top of the mix or replace fine aggregates Rubber content rates range from 5% to 20% of the virgin binder content Aggregate gradation, rubber gradation, binder content and air voids are critical design parameters	Crumb rubber mixed with asphalt binder between 176 °C and 226 °C Agitation and cooking needed for hours or days Rubber and binder separate if not constantly mixed Crumb rubber swells by absorption of maltenes Properties are influenced by base binder composition, blending time, temperature, amount and size of ground tire rubber and the grinding method
*Performance Enhancement Mechanism*	An increase in resistance to rutting due to mechanical interaction Improved resistance to cracking due to flexible behavior of rubber	An increase in viscosity of binder is observed due to absorption of lighter compounds in swelling process The swelling of the crumb rubber adds flexibility to asphalt mix matrix
*Equipment Needs*	Major investment in special equipment in asphalt plant is not needed	Special equipment and handling procedures need significant investment Special chemicals are needed
*Advantages/Drawbacks*	Typically costs 15–50% less than wet process and polymer-modified asphalt binders Asphalt binder in mix should be increased by 0.2 to 0.4%	Separation is a real issue Extended heating time weakens the pore structure in rubber, resulting in the release of maltenes and tire processing oils, reducing the dynamic viscosity of the binder Polymer-modified binders may not be used due to adverse interaction with asphalt rubber

**Table 4:** Comparison of lab performance aspects of dry and wet processes.

Performance Indicator	Dry Process	Wet Process
Lab Performance	5% crumb rubber increased binder viscosity and raised the performance grade by one level Base binder grade was determined to play an important role in observed improvements in PG grades An increase in the amount of rubber resulted in a higher resistance of binder to rutting The negative impact of increasing rubber content on m-values and cracking resistance was found to be small 10% crumb rubber (<1.397 mm) in PG 58-28 increased binder viscosity by 75–80% and bumped the PG grade up to 3 levels Improvement in fatigue performance of PG 58-28 was observed with an addition of 10% rubber Dry process CRM mix performance was competitive with the wet process mix and better than PMA in all key test categories 10 and 15% addition of rubber in a PG 70-22 led to a two-grade increase in high-temperature PG Neither the type of rubber (truck/auto), grinding processes (cryogenic/ambient) nor particle size distribution had a significant impact on the performance of CRM binder Dry process rubber-modified binders exhibited strong similarities to wet process and PMA binders in their respective master curves	Addition of more CR to binders increased the critical low-temperature PG Without properly engineered agitation systems, settling is an issue for wet-blended CRM binders Gap-graded CRM asphalt mixes produced by wet and dry processes resulted in mixes with better fatigue performance compared with unmodified mixes Resilient modulus test data indicated that wet process mixes had higher Mr values, compared to dry process mixes Fatigue performance of the CRM mixes at 20 °C showed the most significant improvement Wet process CRM mixes were stronger than dry mixes in terms of high temperature deformation resistance and tensile strength at ambient temperature Moisture resistance of both dry and wet process mixes was lower than unmodified mix. Addition of hydrated lime was found to improve the resistance of the CRM mixes to moisture damage

**Table 5:** Comparison of field performance aspects of dry and wet processes.

Performance Indicator	Dry Process	Wet Process
Field Performance	The CRM pavements (wet and dry) were more resistant to fatigue and moisture-induced damage than the PMA binders, Wet and dry CRM mixes accumulated approximately 18% less permanent shear strain at 5000 cycles compared to PMA Rut resistances of both dry process rubber and polymer-modified binders were found to be similar Within 30–60 min of mix production, dry process asphalt mimicked the performance of wet process asphalt across a wide range of binder and mix tests. Pavement condition rating system indicated a total expected service life of 17 to 23 years for GTR pavement section. However, sections constructed using PMA mixes had a total expected service life of 12 to 18 years With proper mixing, low and no-cook (dry process) CRM asphalt seemed to perform as well as terminal-blend CRM asphalt, both in the lab and in the field Dry process CRM asphalts exhibited excellent workability, good to excellent compaction, good to excellent minimum compaction temperatures, low field emissions and minimal stickiness during handling, even for rubber asphalt applications. This was true for breakdown temperatures as low as 113 °C.	Comparative evaluations between dry mix CRM asphalts and polymer-modified asphalts (3 lbs. of SBS per 100 lbs. of binder, as low as 6.4 lbs. of rubber per 100 lbs. of binder) and wet mix rubberized asphalt showed no difference in performance in the field, including heavily-travelled interstate highways over periods as long as 1.5 decades The method for rubber introduction into asphalt (wet or dry) did not have a material impact on the quality or performance of the paved surfaces. The CRM pavements (wet and dry) were more resistant to fatigue and moisture-induced damage than the PMA binders The amount of rubber added, temperature, dwell times, ratio of binder to rubber additions and engineering of the rubber appeared to be the most important variables in regard to pavement performance. Wet and dry CRM mixes accumulated approximately 18% less permanent shear strain at 5000 cycles compared to PMA

**Table 6:** State DOT specifications for terminal-blended CRM biners.

State DOT	ADOT PG 76-22 TR+	TxDOT AC-20-5TR	FDOT ARB 5	FDOT ARB 12
Tests on un-aged binder				
Base Asphalt Cement Grade	PG 76-22	AC-20	PG 67-22	PG 67-22
Minimum CRM by Total Weight of Binder (%)	-	5	-	-
Minimum CRM by Weight of Asphalt Cement (%)	9	-	5	12
Minimum Rotational Viscosity (Pascal-seconds)	-	-	0.4 at 150 °C	1.0 at 150 °C
Viscosity (Poise) 60 °C/135 °C (0AASHTO 202)	-	Min 2000/ Max 10.0	-	-
Minimum, Maximum Interaction Temperatures	-	-	150 °C, 170 °C	150 °C, 175 °C
Minimum Interaction Time	-	-	10 min	15 min
G*/sin δ @ 76 °C @ 10 rad/s	Min 1.0 kPa	-	-	-
G*/sin δ @ 64 °C @ 10 rad/s	-	Min 1.0 kPa	-	-
Phase angle, δ	Max 75°	-	-	-
Needle Penetration (0.1 mm) 25 °C 100 g, 5 s	-	75–115	-	-
Softening Point, Minimum (AASHTO T53)	60 °C	49 °C	-	-
Elastic Recovery, 10 °C, Minimum	55%	55%	-	-
Tests on RTFO-aged binder				
Retained Penetration Ratio (% of Original) 25 °C	-	60–100	-	-
G*/sin δ @ 76 °C @ 10 rad/s	Min 2.2 kPa	-	-	-
Tests on PAV-aged binder				
G*/sin δ @ 31 °C @ 10 rad/s	Min 5000 kPa	-	-	-
Creep Stiffness, S @ −12 °C, 60 s	Max 300 MPa	-	-	-
Creep Stiffness, S @ −18 °C	-	Max 300 MPa	-	-
m-value @ −12 °C, 60 s	Min 0.300	-	-	-
m-value @ −18 °C	-	Min 0.300	-	-

**Table 7:** Specifications for on-site blended CRM binders.

State DOT	ADOT Type 1 Binder	ADOT Type 2 Binder	ADOT Type 3 Binder	TxDOT Type I Binder	TxDOT Type II Binder	TxDOT Type III Binder	FDOT ARB 20	Caltrans
Base Asphalt Cement Grade	PG 64–16	PG 58–22	PG 52–28	PG 58–28 PG 64–22	PG 58–28, PG 64–22	PG 58–28, PG 64–22	PG 64–22	AR-4000
Minimum CRM by Total Weight of Binder (%)	-	-	-	15	15	15	-	-
Maximum CRM by Total Weight of Binder (%)	20	20	20	-	-	-	20	18
Modifier Content by Weight of Asphalt Cement (%)	Not Allowed	Not Allowed	Not Allowed	Not Used	Not Used	Not Used	Not Used	-
Minimum, Maximum Interaction Temperatures	163 °C, 190 °C	163 °C, 190 °C	163 °C, 190 °C	-	-	-	170 °C, 190 °C	190 °C, 226 °C
Minimum Interaction Time	60 Minutes	60 Minutes	60 Minutes	-	-	-	30 Min	45 Min
Rotational Viscosity	1.5–4.0 at	1.5–4.0 at	1.5–4.0 at	1.5–5.0 at	1.5–5.0 at	1.5–5.0 at	1.5 at	1.5–4.0 at
Pascal-seconds	177 °C	177 °C	177 °C	175 °C	175°C	175°C	175 °C	190 °C
Penetration 4 °C, 200 g, 60 s (ASTM D 5) 0.1 mm, minimum	10	15	25	-	-	-	-	-
Cone Penetration 25 °C, 150 g, 5 s, 0.1 mm	-	-	-	-	-	-	-	25–70
Needle Penetration (0.1 mm) 25 °C 100 g, 5 s	-	-	-	25–75	25–75	50–100	-	-
Softening Point Minimum (AASHTO T53)	57 °C	54 °C	52 °C	57 °C	54 °C	52 °C	-	52 °C
Softening Point Maximum (AASHTO T53)	-	-	-	-	-	-	-	74 °C
Resilience (%) 25 °C, Minimum (ASTM D 5329)	30	25	20	25	20	10	-	18
Test on RTFO Residue Retained Penetration Ratio (% of Original) 4 °C	-	-	-	75	75	75	-	-
Flash point, C.O.C.	-	-	-	232 °C	232 °C	232 °C	-	-

**Table 8:** Transportation agencies that participated in survey.

No.	Agency	State
1	Alabama Department of Transportation (ALDOT)	AL
2	Alaska Department of Transportation & Public Facilities (ADOT &PF)	AK
3	Arizona Department of Transportation (AZDOT)	AZ
4	Arkansas State Highway and Transportation Department (AHTD)	AR
5	California Department of Transportation (CALTRANS)	CA
6	Colorado Department of Transportation (CDOT)	CO
7	Connecticut Advanced Pavement Lab. (CAP LAB)	CT
8	Delaware Department of Transportation (DelDOT)	DE
9	Department of Transportation (DOT)	NH
10	Florida Department of Transportation (FDOT)	FL
11	Georgia Department of Transportation (GDOT)	GA
12	Iowa Department of Transportation (IowaDot)	IA
13	Kansas Department of Transportation (KDOT)	KS
14	Kentucky Transportation Cabinet (KYTC)	KY
15	Louisiana Department of Transportation & Development (LaDOTD)	LA
16	Maine Department of Transportation (MaineDOT)	ME
17	Maryland State Highway Administration (SHA)	MD
18	Michigan Department of Transportation (MDOT)	MI
19	Minnesota Department of Transportation (MnDOT)	MN
20	Mississippi Department of Transportation (MDOT)	MS
21	Missouri Department of Transportation (MODOT)	MO
22	Montana Department of Transportation (MDT)	MT
23	Nebraska Department of Roads (NDOR)	NE
24	Nevada Department of Transportation (NDOT)	NV
25	New Jersey Department of Transportation (NJDOT)	NJ
26	New Hampshire Department of Transportation (NHDOT)	NH
27	Ohio Department of Transportation (ODOT)	OH
28	Pennsylvania Department of Transportation (PennDOT)	PA
29	Rhode Island Department of Transportation (RIDOT)	RI
30	South Carolina Department of Transportation (SCDOT)	SC
31	Tennessee Department of Transportation (TDOT)	TN
32	Texas Department of Transportation (TxDOT)	TX
33	Utah Department of Transportation (UDOT)	UT
34	West Virginia Division of Highways (WVDOT)	WV
35	Wisconsin Department of Transportation (WisDOT)	WI
36	Washington State Department of Transportation (WsDOT)	WA
37	Vermont Agency of Transportation (VTRANS)	VT
38	Ontario Ministry of Transportation (MTO)	Canada

**Table 9:** Reasons for not using GTR in HMA.

Please Specify the Reason(s) for Not Incorporating GTR in Asphalt Mixes.	
Answer Options	Response Percent
Unsuccessful experience of using GTR in asphalt mixes in the past.	38.9%
Concern over the performance of asphalt mixes containing GTR.	44.4%
Lack of performance data of asphalt mixes containing GTR.	27.8%
Using GTR in asphalt is not cost effective.	61.1%
There is not sufficient incentive to recycling scrap tires in pavement applications.	33.3%
There is not a crumb rubber producer in the state.	22.2%

**Table 10:** Reasons for using GTR in HMA.

What Are the Main Reasons for Using GTR Asphalt Pavements by Your Agency?	
Answer Options	Response Percent
It is cost effective.	25.0%
Better performance compared to conventional materials	66.7%
Significant incentives to recycling scrap tires.	20.8%
Other.	50.0%

**Table 11:** Different Applications of GTR in Pavement.

Please Specify in what Type(s) of Mixes the GTR is Used by Your Agency?	
Answer Options	Response Percent
Hot Mix Asphalt (HMA)	87.0%
Warm Mix Asphalt (WMA)	56.5%
Nonstructural Thin-Lift Overlay (<1.5 in.)	52.2%
Structural Overlays (>1.5 in.)	56.5%
Mill-and-Fill Operation	47.8%
Chip Seal	30.4%
Fog Seal	21.7%
Other	26.1%

**Table 12:** Application of GTR in asphalt mixes based on project type.

Where do You Use Asphalt Mixes Containing GTR (Multiple Answers May Be Selected, if Applicable)?	
Answer Options	Response Percent
Interstate Highways	73.9%
City road	39.1%
State Highway	78.3%
Other (please specify)	30.4%

**Table 13:** Types of processes used by states to incorporate GTR or CR in asphalt mixes.

Please Specify the Type of Process Used by Your Agency in Order to Incorporate GTR or CR in Asphalt Mixes (Please Mark all that Apply and Write in Your Answer if Applicable)?	
Answer Options	Response Percent
Dry Process	13.6%
Wet Process (Terminal Blend)	77.3%
Wet Process (Field Blend)	54.5%

**Table 14:** Availability of guideline/specification/special provision to states incorporating GTR in HMA.

Please Specify if Your Agency Follows a Guideline, Technical Specifications, Special Provision, etc. for Incorporating GTR in Asphalt Mixes?	
Answer Options	Response Percent
No.	13.6%
Yes (Please provide the link to the guideline, technical specifications, special provision, etc.)	86.4%

**Table 15:** Technical considerations for asphalt mixes containing GTR/CR.

What Considerations are Recommended by Your Agency to Be Taken into Account in Design of Asphalt Mixes Containing GTR/CR (Please Mark all that Apply and Write in Your Answer if Applicable)?	
Answer Options	Response Percent
Mix Temperature (Please specify in the comment field).	50.0%
Modification to Binder PG Grade (Please specify in the comment field).	50.0%
Compaction Effort (Please specify in the comment field).	15.0%
Other (Please specify in the comment field).	30.0%

**Table 16:** Criteria used for surface course mixes containing GTR.

Please Specify Test(s) and Criteria Used to Set the Maximum GTR Content (%) Limit in Surface Course.	
Answer Options	Response Percent
Maximum GTR Allowed (%)	95.0%
Test(s) (e.g., fatigue, low temperature cracking, etc.)	70.0%
Criteria (e.g., number of cycles to fatigue failure; creep compliance; indirect tensile strength, etc.)	40.0%

**Table 17:** Criteria used for Base/Intermediate Course Mixes Containing GTR.

Please Specify Test(s) and Criteria Used to Set the Maximum GTR Content (%) Limit in Intermediate/Base Course.	
Answer Options	Response Percent
Maximum GTR Allowed (%)	100.0%
Test(s) (e.g., fatigue, low temperature cracking, etc.)	57.9%
Criteria (e.g., number of cycles to fatigue failure; creep compliance; indirect tensile strength, etc.)	36.8%

**Table 18:** Laboratory tests conducted on asphalt mixes containing GTR.

What Laboratory Performance Tests Are Conducted on Asphalt Mixes Containing GTR (Please Mark all That Apply and Write in Your Answer if Applicable)?	
Answer Options	Response Percent
Rutting (Asphalt Pavement Analyzer or Hamburg Wheel Tracking)	70.6%
Fatigue (Four-Point Bending Beam)	17.6%
Fatigue (Viscoelastic Continuum Damage)	0.0%
Creep Compliance	0.0%
Moisture-Induced Damage (Tensile Strength Ratio or Hamburg Wheel Tracking)	70.6%
Dynamic Modulus, Flow Number, Flow Time	11.8%
Other (please specify)	64.7%

## Data Availability

The data presented in this study are available on request from the corresponding author.
